# Supplementary material for: Breastfeeding support through wet nursing during nutritional emergency: A cross sectional study from Rohingya refugee camps in Bangladesh
Source: PLoS One. 2019 Oct 2;14(10):e0222980. doi: 10.1371/journal.pone.0222980 (PMC6774527; doi:10.1371/journal.pone.0222980)
Supplement: S1 Questionnaire — (PDF) [file pone.0222980.s001.PDF]

Supportive breastfeeding through wet nursing during nutritional emergency: a cross sectional study from the Rohingya refugee camps in Bangladesh

Questionnaire/Participant Code: _______________ Date: ___/___/______

Camp No. / Camp Code: __________________

Block No. / Block Code: __________________

Household/Tent No.: ________________________ Registration No. (If available): ___________________

**Consent to participate**	Written consent was provided (put tick)	**1= Yes**<br>**2= No/ refusal**	Reason(s) for refusal (if applicable):

A. Personal Information

**Q. Code**	**Questions**	**Responses/ categories**	**Remarks (if any)**
A1	Name of wet nurse (write)	_________________________	
A2	Age (years)	_________ years	
A3	Number of family members	_________ members	
A4	Number of children	_________ children	
A5	Age (months) of the youngest child	_________ months	
A6	Do you have husband (put tick)	1= Yes<br>2= No	
A7	Education level of wet nurse (put tick)	1= No education<br>2= Can read and write<br>3= Primary level<br>4= Secondary level<br>5= Above secondary	
A8	Education level of the husband (put tick)	1= No education<br>2= Can read and write<br>3= Primary level<br>4= Secondary level<br>5= Above secondary	

B. Knowledge on IYCF-E

Q. Code	Questions	Responses/ categories	Remarks (if any)
B1	What do you know about the importance of breastfeeding? (listen to the responses and put tick where appropriate)<br><br>[1= if participants can correlate the importance of breastfeeding with the benefit of child, mother, family and society, 2= if participants can at least tell any specific breastfeeding benefit with proper reasoning, 3= if no benefit is told precisely with reasoning]	1= Good knowledge<br>2= Basic knowledge<br>3= Poor knowledge	
B2	What do you know about the importance of breastfeeding in emergency? (listen to the responses and put tick where appropriate)<br><br>[1= if participants can correlate the breastfeeding with other fatal exposures regarding formula and bottle feeding, 2= if participants can tell the other benefits with reasoning, 3= if no specific benefit is told with proper reasoning]	1= Good knowledge<br>2= Basic knowledge<br>3= Poor knowledge	
B3	Knowledge on early initiating the breastfeeding; when breastfeeding to be started? (listen to the responses and put tick where appropriate)<br><br>[1= within 1 hour of birth and no pre-lacteal food, 2= the sooner the better but no specific time frame, 3= no specific answer is told]	1= Good knowledge<br>2= Basic knowledge<br>3= Poor knowledge	
B4	How long a child should breastfed exclusively?	_____(months/years)	
B4.1	Why is exclusive breastfeeding important?(put down the response)	___________________	
B5	At least how long a child should be continued with breastfeeding?	______(months/years)	
B5.1	Why is it important? (put down the answer)	___________________	
B6	From where did you learn the breastfeeding messages? (trick multiple if applicable)<br><br>**[Community leaders:** Majhi, Imam, Female religious leaders<br>**Service providers:** Community health and nutrition workers (CHNW), nutrition or health professionals, other relevant facilities]	1= Family/ relatives<br>2= Teachers<br>3= Community leaders<br>4= Service providers<br>5= Other (mention)	

C. Information about wet nursing

Q. Code	Questions	Responses/ categories	Remarks (if any)
C1	Did you ever hear about wet nursing before arriving in the camp? (Put tick)	1= Yes<br>2= No	
C2	How long have you performed as wet nurse?	___________ (month/year)	

Q. Code	Questions	Responses/ categories	Remarks (if any)
C3	Till now how many children did you support through wet nursing?	___________ children	
C4	Age of the child when you started breastfeeding. (consider the current or most recent child)	___________ months	
C4.1	Sex of the child	1= Boy<br>2= Girl	
C4.2	Any family relationship with child	1= Yes<br>2= No	
C4.3	Why the breastfeeding support was essential?	1= Death of biological mother<br>2= Mother was unable to breastfeed<br>3= Other (mention in remarks)	
C4.4	Who did inform you about the baby?<br><br>**[Community leaders:** Majhi, Imam, Female religious leaders<br>**Service providers:** Community health or nutrition workers (CHNW), nutrition or health professionals, other relevant facilities]	1= Family members<br>2= Relatives<br>3= Community leaders<br>4= Service providers<br>5= NGOs (other than IYCF-E)	
C4.5	Are you currently breastfeeding the baby?	1= Yes<br>2= No	
C4.6	How long (month) did you breastfeed the child?	___________months	
C5	How did you know about wet nursing?<br><br>**[Community leaders:** Majhi, Imam, Female religious leaders<br>**Service providers:** Community health or nutrition workers (CHNW), nutrition or health professionals, other relevant facilities]	1= Family/ relatives<br>2= Teacher<br>3= Community/ religious leaders<br>4= Service providers<br>5= Others (mention in remarks)	
C6	How did you get involve into wet nursing?(select multiple if applicable)<br><br>**[Community leaders:** Majhi, Imam, Female religious leaders<br>**Service providers:** Community health or nutrition workers (CHNW), nutrition or health professionals, other relevant facilities]	1= Family/ relatives' initiatives<br>2= Teachers' initiatives<br>3= Community leaders' initiatives<br>4= Service providers' initiatives<br>5= Other (mention in remarks)	
C6.1	If the answer is 4, then how frequently did the CHNW/NGO follow-up you?	1= Everyday<br>2= Every week<br>3= Monthly<br>4= Never after	
C7	Did you need to require permission from your family members?	1= Yes<br>2= No	
C7.1	If "Yes", who provided permission?	1= Husband<br>2= Other family member<br>3= Both	
C8	How many times a day did you breastfeed as wet nurse?	____________times a day	
C9	How long (minutes/hours) was the every breastfeeding session?	____________ minutes/ hours	

Q. Code	Questions	Responses/ categories	Remarks (if any)
C10	Did you breastfeed overnight?	1= Yes<br>2= No	
C11	Did you receive any gift/incentive for wet nursing?(Put tick)	1= Yes<br>2= No	
C11.1	If "Yes", what type of remuneration/ gift/ incentive was it? (mention)	________________________	
C11.2	Who provided you the remuneration/ gift/ incentive?	1= Child's family<br>2= NGO/ IYCF-E partners<br>3= Others (mention in remarks)	
C11.3	Will you agree to perform as wet nurse in future without any incentive?	1= Yes<br>2= No<br>3= Will be happy if given	
C12	Did you face any problem while wet nursing?(Put tick)	1= Yes<br>2= No	
C12.1	If "Yes", what type of problem was it? (tick multiple if required)	1= Constrain from own family<br>2= Misunderstanding with child's family<br>3= Time constrain<br>4= Disrupted my personal routine<br>5= Distance of child's house<br>6= Negative community perception<br>7= Others (mention in remarks)	
C12.2	If "Yes", to what extent the problem was?	1= Manageable<br>2= Often problematic<br>3= Serious problematic	
C12.3	If misunderstanding was happened with child's relatives/family, how often was it?	1= Rarely<br>2= Often<br>3= Very often	
C13	Did you face any physical or mental problem while wet nursing? (Put trick)	1= Yes<br>2= No	
C13.1	If "Yes", what type of problem was it?	2= Weakness<br>3= Mental stress<br>4= Inadequate milk secretion<br>5= Others (mention)____________	
C14	How did you solve the problems? (choose multiple if required)	1= Mutual understanding<br>2= Supports from CHNW/NGO<br>3= Community or religious leaders' support<br>4= Could not solve yet	
C15	Why/when did you stop performing as wet nurse? (if applicable)	1= Beast feeding was completed<br>2= Misunderstanding with child's family<br>3= Personal unwillingness<br>4= Other (mention) _________	
C16	Did you ever cease breastfeeding?	1= Yes<br>2= No	

Q. Code	Questions	Responses/ categories	Remarks (if any)
C16.1	If "Yes", how many years/months ago?	____________ (years/months)	
C16.2	If "Yes", how has the re-lactation been possible? (select multiple if required)	1= Counseling and motivation by nutrition service providers or specialists<br>2= Family support<br>3= Self motivation<br>4= Others (mention) __________	
C17	What was the best motivation for you to perform as wet nurse?	1= Self-motivation<br>2= Counseling and motivation by service providers (CHNW)<br>3= Religious inspiration<br>4= Family supports<br>5= Incentives or gifts<br>6= Others	
C18	What was your overall experience after performing as a wet nurse?	1= Very good<br>2= Good<br>3= Normal<br>4= Bad<br>5= Very bad	

D. Nutritional status

Q. Code	Questions	Response/ category/ value	Remarks (if any)
D1	MUAC	______________(cm/mm)	
D2	Body height	______________(meters)	
D3	Body weight	______________(kg)	

Give thanks to the participant. Kindly re-check the questionnaire so that all the information is collected accordingly before leaving the household/tent.

Additional comment for the interviewer (if any):

______________________________ ______________________________

Signature of the interviewer Signature of the data investigator
