# Supplementary material for: Breastfeeding support through wet nursing during nutritional emergency: A cross sectional study from Rohingya refugee camps in Bangladesh
Source: PLoS One. 2019 Oct 2;14(10):e0222980. doi: 10.1371/journal.pone.0222980 (PMC6774527; doi:10.1371/journal.pone.0222980)
Supplement: S2 Questionnaire — (PDF) [file pone.0222980.s002.pdf]

পুষ্টি সম্পর্কিত জরুরী অবস্থায় ধাত্রীর মাধ্যমে দুধপান সহায়তা প্রদান: রোহিঙ্গা শরনার্থী ক্যাম্প থেকে একটি ক্রস-সেকসনাল স্ট্যাডি

প্রশ্নাবলী/অংশগ্রহনকারী কোড ঃ ____________________ তারিখঃ ____/______/_______

ক্যাম্প নং/কোডঃ _____ ______________________

ব্লক নং/কোডঃ ______________________________

বাড়ি/তাবু নংঃ ______________________________ রেজিস্ট্রেশন নং (যদি থাকে)ঃ ________________________

সাক্ষাৎকার প্রদান/ অংশগ্রহন করতে সম্মতি	অংশগ্রহনকারীর লিখিত সম্মতি নেওয়া হয়েছে কিনা (টিক চিহ্ন দিন)	১= হ্যাঁ<br>২= না/প্রত্যাখ্যান	প্রত্যাখ্যানের কারণ (যদি থাকে)ঃ

ক. সাধারণ তথ্য

প্রশ্ন কোড	প্রশ্ন	উত্তর	মন্তব্য
ক১	ধাত্রীর নাম		
ক২	ধাত্রীর বয়স (বছর)		
ক৩	ধাত্রীর পরিবারের সদস্য সংখ্যা		
ক৪	ধাত্রীর সন্তান সংখ্যা		
ক৫	ধাত্রীর কনিষ্ঠ সন্তানের বয়স (মাস)		
ক৬	ধাত্রীর স্বামী আছে কি না (টিক চিহ্ন দিন)	১= হ্যাঁ<br>২= না	
ক৭	ধাত্রীর শিক্ষাগত যোগ্যতা (টিক চিহ্ন দিন)	১= নিরক্ষর<br>২= লিখতে ও পড়তে পারে<br>৩= প্রাথমিক ধাপ<br>৪= মাধ্যমিক ধাপ<br>৫= মাধ্যমিকোত্তর	
ক৮	স্বামীর শিক্ষাগত যোগ্যতা (টিক চিহ্ন দিন)	১= নিরক্ষর<br>২= লিখতে ও পড়তে পারে<br>৩= প্রাথমিক ধাপ<br>৪= মাধ্যমিক ধাপ<br>৫= মাধ্যমিকোত্তর	

খ. আইওয়াইসিএফ-ই বিষয়ক জ্ঞান

প্রশ্ন কোড	প্রশ্ন	উত্তর	মন্তব্য
খ১	বুকের দুধ খাওয়ানোর গুরুত্ব/উপকারীতা সম্পর্কে জ্ঞান। (উত্তর শুনুন, যাচাই করুন এবং টিক চিহ্ন দিন)<br><br>[১ = যদি অংশগ্রহনকারী বুকের দুধ খাওয়ানো সাথে শিশু, মা, পরিবার ও সমাজের উপকারীতা সম্পর্কে বলতে পারে, ২ = অংশগ্রহনকারীর যদি অন্যান্য উপকারীতা যুক্তিসহ বলতে পারে, ৩ = যদি নির্দিষ্ঠ করে যুক্তিসহ কিছু বলতে না পারে]	১= সম্যক ধারণা<br>২= মৌলিক ধারণা<br>৩= স্বল্প ধারণা	
খ২	জরুরী অবস্থায় বুকের দুধ খাওয়ানোর গুরুত্ব/উপকারীতা সম্পর্কে জ্ঞান। (উত্তর শুনুন, যাচাই করুন এবং টিক চিহ্ন দিন)<br><br>[১ = অংশগ্রহনকারী যদি বুকের দুধ না খাওয়ানো কিংবা গুড়ো দুধ খাওয়ানোর ফলে সৃষ্ট স্বাস্থ্য ঝুঁকির কথা বলতে পারে, ২ = যদি অন্যান্য উপকারের কথা যুক্তিসহ বলতে পারে, ৩ = যদি নির্দিষ্ট কোন উপকারের কথা যুক্তিসহ বলতে না পারে]	১= সম্যক ধারণা<br>২= মৌলিক ধারণা<br>৩= স্বল্প ধারণা	
খ৩	জন্মের পর বুকের দুধ খাওয়ানো শুরু করা সম্পর্কিত জ্ঞান- জন্মের পর কখন শিশুকে বুকের দুধ খাওয়ানো শুরু করা উচিত? (উত্তর শুনুন, যাচাই করুন এবং টিক চিহ্ন দিন)<br><br>[১ = জন্মের ১ ঘন্টার মধ্যে বুকের দুধ খাওয়ানো এবং অন্য কোন খাবার না দেওয়া, ২ = যত আগে দিবে তত ভাল কিন্তু কোন নির্দিষ্ট কোন সময় উল্লেখ না করা, ৩ = নির্দিষ্ট করে কিছু বলতে না পারা]	১= সম্যক ধারণা<br>২= মৌলিক ধারণা<br>৩= স্বল্প ধারণা	
খ৪	জন্মের পর কত সময় পর্যন্ত শিশুকে শুধুমাত্র বুকের দুধ খাওয়ানো উচিত?	______(দিন/মাস/বছর)	
খ৪.১	৬ মাস বয়স পর্যন্ত শিশুকে শুধুমাত্র বুকের দুধ খাওয়ানোর উপকারীতা কী? (উত্তর লিখুন)		
খ৫	অন্তত কত সময় পর্যন্ত শিশুকে বুকের দুধ খাওয়ানো চালিয়ে যাওয়া উচিত?	______(দিন/মাস/বছর)	
খ৫.১	ইহা কেন জরুরী? (উত্তর লিখুন)		
খ৬	আপনি কোথায়/কিভাবে শিশুকে বুকের দুধ খাওয়ানো সম্পর্কে জেনেছেন? (প্রয়োজনে একাধিক টিক চিহ্ন দিন)<br><br>[কমিউনিটি প্রতিনিধিঃ মাঝি, ইমাম, মহিলা ধর্মীয় নেতা<br>স্বাস্থ্য/পুষ্টি সেবাদানকারীঃ কমিউনিটি স্বাস্থ্য/পুষ্টি কর্মী কিংবা বিশেষজ্ঞ কিংবা অন্য সেবাবে দ্র]	১= পারিবার/ আত্মীয়<br>২= শিক্ষক<br>৩= কমিউনিটি প্রতিনিধি<br>৪= স্বাস্থ্য/পুষ্টি সেবাদানকারী<br>৫= অন্যান্য (উল্লেখ করুন)	

ধাত্রী কর্তৃক বুকের দুধ খাওয়ানো সম্পর্কিত তথ্য

প্রশ্ন কোড	প্রশ্ন	উত্তর	মন্তব্য
গ১	ক্যাম্পে আসার পূর্বে আপনি কি ওয়েট নার্সিং সম্পর্কে অবগত ছিলেন? (টিক চিহ্ন দিন)	১=হ্যাঁ<br>২=না	

প্রশ্ন কোড	প্রশ্ন	উত্তর	মন্তব্য
গ২	আপনি কত সময় ধরে ধাত্রী হিসেবে কাজ/সহযোগিতা প্রদান করছেন?	________(মাস/বছর)	
গ৩	ধাত্রী হিসেবে এ পর্যন্ত কতজন শিশুকে আপনি বুকের দুধ পান করিয়েছেন?	________জন শিশু	
গ৪	শিশুর কত মাস বয়স থেকে আপনি ধাত্রী হিসেবে তাকে দুধ পান করাচ্ছেন? (যেই শিশুকে সর্বশেষ/বর্তমানে দুধ পান করিয়েছেন/ করাচ্ছেন তাকে বিবেচ্য)	________মাস	
গ৪.১	শিশুর লিঙ্গ।	১=ছেলে<br>২=মেয়ে	
গ৪.২	শিশুর সাথে আপনার পারিবারিক সম্পর্ক আছে/ছিল কি?	১= হ্যাঁ<br>২= না	
গ৪.৩	শিশুকে দুধ পান সহায়তা কেন প্রয়োজন ছিল?	১= শিশুর মায়ের মৃত্যু<br>২= মা শিশুকে দুধ খাওয়াতে অক্ষম<br>৩= অন্যান্য কারণে (উল্লেখ করুন)	
গ৪.৪	শিশুটি সম্পর্কে কে আপনাকে অবগত করেছে?<br><br>[কমিউনিটি প্রতিনিধিঃ মাঝি, ইমাম, মহিলা ধর্মীয় নেতা<br>স্বাস্থ্য/পুষ্টি সেবাদানকারীঃ কমিউনিটি স্বাস্থ্য/পুষ্টি কর্মী কিংবা বিশেষজ্ঞ কিংবা অন্য সেবাকেন্দ্র]	১= পারিবারিক সদস্য<br>২= আত্মীয়<br>৩= রোহিঙ্গা কমিউনিটি প্রতিনিধি<br>৪= স্বাস্থ্য/পুষ্টি সেবাদানকারী<br>৫= অন্য এনজিও (আইওয়াইসিএফ-ই ব্যতীত)	
গ৪.৫	আপনি কি বর্তমানে শিশুটিকে বুকের দুধ খাওয়াচ্ছেন?	১= হ্যাঁ<br>২= না	
গ৪.৬	আপনি কত মাস ধরে শিশুটিকে বুকের দুধ খাওয়াচ্ছেন?	________মাস	
গ৫	আপনি কিভাবে ওয়েট নার্সিং সম্পর্কে অবগত হয়েছেন?<br><br>[কমিউনিটি প্রতিনিধিঃ মাঝি, ইমাম, মহিলা ধর্মীয় নেতা<br>স্বাস্থ্য/পুষ্টি সেবাদানকারীঃ কমিউনিটি স্বাস্থ্য/পুষ্টি কর্মী কিংবা বিশেষজ্ঞ কিংবা অন্য সেবাকেন্দ্র]	১= পারিবার/আত্মীয়<br>২= শিক্ষক<br>৩= রোহিঙ্গা কমিউনিটি প্রতিনিধি<br>৪= স্বাস্থ্য/পুষ্টি সেবাদানকারী<br>৫= অন্যান্য (উল্লেখ করুন)	
গ৬	আপনি কিভাবে/ কার মাধ্যমে ওয়েট নার্স বা ধাত্রী হিসেবে কাজ শুরু করেছেন?<br><br>**কমিউনিটি প্রতিনিধিঃ** মাঝি, ইমাম, মহিলা ধর্মীয় নেতা<br>**স্বাস্থ্য/পুষ্টি সেবাদানকারীঃ** কমিউনিটি স্বাস্থ্য/পুষ্টি কর্মী কিংবা বিশেষজ্ঞ	১= পারিবার/আত্মীয়<br>২= শিক্ষক<br>৩= রোহিঙ্গা কমিউনিটি প্রতিনিধি<br>৪= স্বাস্থ্য/পুষ্টি সেবাদানকারীর মাধ্যমে<br>৫= অন্যান্য (উল্লেখ করুন)	
গ৬.১	উত্তর ৪ হলে, কমিউনিটি স্বাস্থ্য/পুষ্টি সেবাদানকারী আপনার সাথে কিভাবে যোগাযোগ করত?	১= প্রতিদিন<br>২= প্রতি সপ্তাহে<br>৩= প্রতি মাসে<br>৪= পরবর্তীতে যোগাযোগ করেনি	

প্রশ্ন কোড	প্রশ্ন	উত্তর	মন্তব্য
গ৭	ওয়েট নার্স বা ধাত্রী হিসেবে কাজ করার জন্যে আপনাকে পরিবারের কারও কাছ থেকে অনুমতি নিতে হয়েছে কি?	১= হ্যাঁ<br>২= না	
গ৭.১	হ্যাঁ হলে, কার কাছ থেকে অনুমতি নিতে হয়েছে?	১= স্বামী<br>২= অন্যান্য সদস্য<br>৩= উভয়ই	
গ৮	ধাত্রী হিসেবে দিনে কতবার আপনি শিশুকে বুকের দুধ পান করান/করিয়েছেন?	দৈনিক ____________ বার	
গ৯	প্রতিবার কতক্ষণ সময় ধরে দুধ পান করান/করিয়েছেন?	___________ মিনিট/ঘন্টা	
গ১০	ধাত্রী হিসেবে আপনি শিশুকে কি রাতে দুধ পান করান/করিয়েছেন?	১= হ্যাঁ<br>২= না	
গ১১	ধাত্রী হিসেবে কাজ করার জন্যে আপনি কি কোন পুরস্কার/সম্মানী গ্রহণ করেছেন? (টিক চিহ্ন দিন)	১= হ্যাঁ<br>২= না	
গ১১.১	হ্যাঁ হলে, কী ধরণের পুরস্কার/সম্মানী গ্রহণ করেছেন? (উল্লেখ করুন)		
গ১১.২	হ্যাঁ হলে, কার কাছ থেকে আপনি পুরস্কার/সম্মানী গ্রহণ করেছেন?	১= শিশুর পরিবার<br>২= এনজিও/ আইওয়াইসিএফ-ই সহযোগী<br>৩= অন্যান্য (উল্লেখ করুন)	
গ১১.৩	আপনি কি ভবিষ্যতে কোন পুরস্কার/সম্মানী ব্যতীত ধাত্রী হিসেবে সহযোগিতা করতে আগ্রহী?	১= হ্যাঁ<br>২= না<br>৩= পেলে খুশি হব	
গ১২	ধাত্রী হিসেবে কাজ করার ক্ষেত্রে আপনি কি কোন সমস্যার মুখোমুখি হয়েছেন ? (টিক চিহ্ন দিন)	১= হ্যাঁ<br>২= না	
গ১২.১	হ্যাঁ হলে,কী ধরণের সমস্যা ছিল? (প্রয়োজনে একাধিক উত্তর বাছাই করুন)	১= নিজ পারিবারিক সমস্যা<br>২= শিশুর পারিবারের সাথে ভুল বোঝাবুঝি<br>৩= সময় স্বল্পতা<br>৪= নিজস্ব কাজে ব্যাঘাত ঘটা<br>৫= শিশুর বাড়ির দুরত্ব<br>৬= নেতিবাচক সামাজিক ধারণা<br>৭= অন্যান্য (উল্লেখ করুন)	
গ১২.২	হ্যাঁ হলে, সমস্যার তীব্রতা কেমন ছিল?	১= সহজে সমাধানযোগ্য<br>২= মোটামুটি কঠিন<br>৩= অত্যন্ত কঠিন	

প্রশ্ন কোড	প্রশ্ন	উত্তর	মন্তব্য
গ১২.৩	উত্তর ২ হলে, শিশুর পরিবারের সাথে কেমন/কত সময় পরপর ভুল বুঝাবুঝি হয়/হয়েছে?	১= কদাচিৎ<br>২= প্রায়শ<br>৩= খুব বেশি	
গ১৩	ধাত্রী হিসেবে কাজ করতে গিয়ে আপনি কি কোন শারীরিক বা মানসিক সমস্যার সম্মুখীন হয়েছেন?	১= হ্যাঁ<br>২= না	
গ১৩.১	হ্যাঁ হলে, কী ধরনের সমস্যা ছিল?	১= দুর্বলতা<br>২= মানসিক চাপ<br>৩= অপর্যাপ্ত দুধ নিঃসরণ<br>৪= অন্যান্য (উল্লেখ করুন)	
গ১৪	আপনি কিভাবে সমস্যাগুলোর সমাধান করেছেন? (প্রয়োজনে একাধিক উত্তর বাছাই করুন)	১= শিশুর পরিবার/আত্মীয়ের সাথে পারস্পরিক আলোচনা/পরামর্শ<br>২= কমিউনিটি স্বাস্থ্য/পুষ্টি কর্মীর সহায়তা/ পরামর্শ<br>৩= কমিউনিটি/ধর্মীয় নেতার সহায়তা<br>৪= সমস্যার সমাধান করা যায়নি	
গ১৫	ধাত্রী হিসেবে আপনি কখন/কেন বুকের দুধ খাওয়ানো বন্ধ করেছেন? (প্রযোজ্য ক্ষেত্রে)	১= শিশুকে যথাযত সময় পর্যন্ত বুকের দুধ খাওয়ানো সম্পন্ন হয়েছে<br>২= শিশুর পরিবার/আত্মীয়ের সাথে সম্পর্ক খারাপ হয়েছে<br>৩= ব্যক্তিগত অনিচ্ছা<br>৪= অন্যান্য কারণ (উল্লেখ করুন)	
গ১৬	পূর্বে আপনি কি কখনো পুরোপরি বুকের দুধ খাওয়ানো ছেড়ে দিয়েছিলেন?	১= হ্যাঁ<br>২= না	
গ১৬.১	হ্যাঁ হলে, কত মাস পূর্বে?	______ মাস	
গ১৬.২	হ্যাঁ হলে, পুনরায় দুধ খাওয়ানো কিভাবে সম্ভব হল? (প্রয়োজনে একাধিক উত্তর বাছাই করুন)	১= স্বাস্থ্য/পুষ্টি কর্মী বা বিশেষজ্ঞের প্রেরণা ও পরামর্শ<br>২= পরিবার কর্তৃক মানসিক সহায়তা<br>৩= ব্যক্তিগত অনুপ্রেরণা<br>৪= অন্যান্য (উল্লেখ করুন)	
গ১৭	ধাত্রী হিসেবে কাজ করার ক্ষেত্রে আপনার জন্যে সর্বোচ্চ অনুপ্রেরণা কী ছিল?	১= ব্যক্তিগত অনুপ্রেরণা<br>২= কমিউনিটি স্বাস্থ্য/পুষ্টি কর্মী কর্তৃক প্রেরণা ও পরামর্শ<br>৩= ধর্মীয় প্রেরণা<br>৪= পারিবারিক সহায়তা<br>৫= পুরস্কার/সম্মানী<br>৬= অন্যান্য (উল্লেখ করুন)	

প্রশ্ন কোড	প্রশ্ন	উত্তর	মন্তব্য
গ১৮	ধাত্রী হিসেবে কাজ করতে পেরে বা দুধপানে সহায়তা করতে পেরে আপনার কেমন অভিজ্ঞতা হয়েছে?	১= খুব ভাল<br>২= ভাল<br>৩= স্বাভাবিক<br>৪= খারাপ<br>৫= খুব খারাপ	

ঘ. পুষ্টিগত অবস্থা

প্রশ্ন কোড	প্রশ্ন	উত্তর/মান	মন্তব্য
ঘ১	মুয়াক	____________(সে.মি./মি.মি)	
ঘ২	দৈহিক উচ্চতা	____________(মিটার)	
ঘ৩	দৈহিক ওজন	____________(কেজি)	

অনুগ্রহ করে প্রশ্নপত্রের সকল তথ্য সঠিকভাবে সংগ্রহ করা হয়েছে কিনা তা পুর্নযাচাই করুন, পরিশেষে অংশগ্রহনকারীকে ধন্যবাদ জানান এবং সাক্ষাৎকারটি সমাপ্ত করুন।

সাক্ষাৎকার গ্রহনকারী কর্তৃক অতিরিক্ত কোন মন্তব্য (যদি থাকে):

____

সাক্ষাৎকার গ্রহনকারীর স্বাক্ষর

____

উপাত্ত নিরীক্ষকের স্বাক্ষর
