# Supplementary material for: Breastfeeding support through wet nursing during nutritional emergency: A cross sectional study from Rohingya refugee camps in Bangladesh
Source: PLoS One. 2019 Oct 2;14(10):e0222980. doi: 10.1371/journal.pone.0222980 (PMC6774527; doi:10.1371/journal.pone.0222980)
Supplement: Attachment — Submitted filename: Response to Reviewers.docx [file pone.0222980.s004.docx]

31 August 2019

Dr. Joerg Heber

Editor-in-Chief, PLOS ONE

And

## Dr. Russell Kabir

## Academic Editor, PLOS ONE

## Subject: Rebuttal letter for the revised manuscript titled “Breastfeeding support through wet nursing during nutritional emergencies: a cross sectional study from Rohingya refugee camps in Bangladesh” with submission ID: [PONE-D-19-16700] - [EMID: c63b02e88307dd9d]

Dear Sir,

Thank you for your e-mail on 2 August 2019 enclosing editor’s and reviewers’ comments. We have carefully reviewed the comments and have revised the manuscript accordingly. Our responses are given in a point-by-point manner below.

We, therefore, hope that the revised manuscript has now quality to meet the criteria for publications. However, we are ready to address any further issue raised by editor and reviewers. We look forward to hearing from you.

Best regards,

M. A. Rifat

Corresponding author

E-mail: [rifatahmed011@gmail.com](mailto:rifatahmed011@gmail.com)

Phone: +8801684-774313

**Response to editor’s comments:**

Thank you for your valuable comments. We have addressed each of your points below:

1. The revised manuscript has been prepared as PLOS ONE's style requirements, including those for file naming.
2. Questionnaire (both in English and Bengali version) used in this study has been provided as “Supporting Information”.
3. Copyediting of manuscript for language usage, spelling, and grammar has been accomplished with the help of professional English editors from Editage ([www.editage.com](http://www.editage.com)). Thanks for your guidance in this regard.
4. A copy of the manuscript showing the changes by highlighting them has been uploaded as a “Supporting Information” file. The edited manuscript has been provided as “Manuscript”.
5. Methods section has been revised accordingly. The demographic and geographic perspective has been addressed. Data collection and data analysis methods have been elaborated for better understanding. Justification of small sample size has been provided. How the questionnaire was pre-tested and validated has been addressed.
6. Data availability statement has been revised and “Minimal Underlying Dataset” has been provided.

**Response to the comments from Reviewer-1:**

Thank you very much for appreciating our work. We highly care your comments thus provided the best effort to address the issues as following:

1. The title has been revised by professional English editor and has been changed to “Breastfeeding support through wet nursing during nutritional emergencies: a cross sectional study from Rohingya refugee camps in Bangladesh”.
2. The short title has also been revised and has been changed to “Breastfeeding support through wet nursing”.
3. Considering the comment from you as well as academic editor we have made the manuscript revise by professional English editing experts.
4. There are very few existing literatures regarding wet nursing in refugee context. However, we have focused few examples of wet nursing (such as in Ellembelle Nzema, Ghana and in Turkey) in the discussion section correlating to our findings. In the revised introduction section we mainly focused on the current subject context.
5. Justification of small sample size has been provided in Methods section as well as in Discussion section showing the evidence of available literature.
6. Associations from linear regression have been explained in Discussion section showing other evidences from literature.
7. Significance level (P) at measures of association has been mentioned.

**Response to the comments from Reviewer-2:**

Thanks for your valuable comments which helped us to improve the manuscript quality. We have revised the manuscript as per your comments as following:

1. We have carefully revised the tables, statistical units, and units of measurements in the appropriate places.
2. In table 1, “n” has been mentioned. 95% CI has been mentioned in Table 3. To mention the age, the term “month” has been used instead of “m”.
3. In tables and figures, terminologies and grammatical issues have been corrected accordingly.

**Response to the comments from Reviewer-3:**

We are glad to hear the appreciation of our work from you. Thank for adding value to our work through your valuable comments. However, this is how we considered your comments and revised the manuscript:

1. The manuscript has been edited by professional English editors.
2. Justification of small sample size has been discussed in methodology and discussion section which are highlighted.
3. Some recent evidence of wet nursing has been cited in discussion section. The discussion section has been revised with more evidences from literatures.
4. Under-nutrition prevalence in the camp was provided to portray the aggravating factors in the camps. However, some part of this discussion has been replaced to the methodology section (study setting) for better understanding.

We highly appreciate your comments and look forward to hearing from you regarding the revised manuscript. Thank you from our team.
